# Bacterial enrichment prior to third-generation metagenomic sequencing improves detection of BRD pathogens and genetic determinants of antimicrobial resistance in feedlot cattle

**DOI:** 10.3389/fmicb.2024.1386319

**Published:** 2024-05-08

**Authors:** Emily K. Herman, Stacey R. Lacoste, Claire N. Freeman, Simon J. G. Otto, E. Luke McCarthy, Matthew G. Links, Paul Stothard, Cheryl L. Waldner

**Affiliations:** ^1^Department of Agricultural, Food, and Nutritional Science, Faculty of Agricultural, Life, and Environmental Sciences, University of Alberta, Edmonton, AB, Canada; ^2^Department of Large Animal Clinical Sciences, Western College of Veterinary Medicine, University of Saskatchewan, Saskatoon, SK, Canada; ^3^HEAT-AMR (Human-Environment-Animal Transdisciplinary AMR) Research Group, School of Public Health, University of Alberta, Edmonton, AB, Canada; ^4^Healthy Environments Thematic Area Lead, Centre for Healthy Communities, School of Public Health, University of Alberta, Edmonton, AB, Canada; ^5^Department of Animal and Poultry Science, College of Agriculture and Bioresources, University of Saskatchewan, Saskatoon, SK, Canada; ^6^Department of Computer Science, College of Arts and Science, University of Saskatchewan, Saskatoon, SK, Canada

**Keywords:** long-read metagenomic sequencing, bovine respiratory disease, antimicrobial resistance, feedlot cattle, antimicrobial resistance genes

## Abstract

**Introduction:**

Bovine respiratory disease (BRD) is one of the most important animal health problems in the beef industry. While bacterial culture and antimicrobial susceptibility testing have been used for diagnostic testing, the common practice of examining one isolate per species does not fully reflect the bacterial population in the sample. In contrast, a recent study with metagenomic sequencing of nasal swabs from feedlot cattle is promising in terms of bacterial pathogen identification and detection of antimicrobial resistance genes (ARGs). However, the sensitivity of metagenomic sequencing was impeded by the high proportion of host biomass in the nasal swab samples.

**Methods:**

This pilot study employed a non-selective bacterial enrichment step before nucleic acid extraction to increase the relative proportion of bacterial DNA for sequencing.

**Results:**

Non-selective bacterial enrichment increased the proportion of bacteria relative to host sequence data, allowing increased detection of BRD pathogens compared with unenriched samples. This process also allowed for enhanced detection of ARGs with species-level resolution, including detection of ARGs for bacterial species of interest that were not targeted for culture and susceptibility testing. The long-read sequencing approach enabled ARG detection on individual bacterial reads without the need for assembly. Metagenomics following non-selective bacterial enrichment resulted in substantial agreement for four of six comparisons with culture for respiratory bacteria and substantial or better correlation with qPCR. Comparison between isolate susceptibility results and detection of ARGs was best for macrolide ARGs in *Mannheimia haemolytica* reads but was also substantial for sulfonamide ARGs within *M. haemolytica* and *Pasteurella multocida* reads and tetracycline ARGs in *Histophilus somni* reads.

**Discussion:**

By increasing the proportion of bacterial DNA relative to host DNA through non-selective enrichment, we demonstrated a corresponding increase in the proportion of sequencing data identifying BRD-associated pathogens and ARGs in deep nasopharyngeal swabs from feedlot cattle using long-read metagenomic sequencing. This method shows promise as a detection strategy for BRD pathogens and ARGs and strikes a balance between processing time, input costs, and generation of on-target data. This approach could serve as a valuable tool to inform antimicrobial management for BRD and support antimicrobial stewardship.

## 1 Introduction

Bovine respiratory disease (BRD) is an important cause of morbidity and mortality and is responsible for most of the injectable antimicrobial use in feedlot cattle in western Canada (Brault et al., 2019). Respiratory disease is complex and multifactorial, often involving a combination of bacterial and viral pathogens exacerbated in animals stressed by weaning, transportation, and comingling (Griffin et al., 2010). Antimicrobial use (AMU) is necessary for managing the impacts of BRD on animal health and welfare. Recent WHO guidelines recommended antimicrobial prescriptions for livestock on diagnostic test data (Aidara-Kane et al., 2018).

Laboratory diagnostics to inform AMU for BRD have traditionally relied on bacterial culture, with or without antimicrobial susceptibility testing (AST). Culture-based diagnostic strategies can take up to a week to provide actionable information and, therefore, have limited utility to guide rapid therapeutic decisions that are critical for ensuring BRD treatment success (Wolfger et al., 2015). Typically, culture-based methods test susceptibility of a single isolate per sample and might not represent the susceptibility status of the population of all bacteria of interest within the sample. Additionally, multi-drug-resistant strains of BRD pathogens have emerged (Michael et al., 2012; Lubbers and Hanzlicek, 2013; Klima et al., 2016, 2020; Rainbolt et al., 2016; Snyder et al., 2017), highlighting the importance of identifying bacterial BRD pathogens and characterizing antimicrobial resistance (AMR) to inform prudent AMU.

Molecular methods have been used in veterinary diagnostic testing for decades and provide comparatively faster and potentially more sensitive results than traditional culture (Loy, 2020). Quantitative polymerase chain reaction (qPCR) is widely used in BRD diagnostic testing in the form of commercially available kits (Pneumo4, DNA Diagnostic A/S, Risskov, Denmark) and has been used for detection and quantitation of antimicrobial resistance genes (ARG) in the nasopharyngeal microbiota of Canadian feedlot cattle (Holman et al., 2018; Guo et al., 2020). While qPCR can be superior to classical culture-based methods in terms of turnaround time, it is inherently limited in scope to the known assay targets for which primers have been developed and tested. Typically, multiple reactions are required to test for the presence of multiple pathogens and ARGs.

In contrast, whole genome sequencing (WGS) produces high resolution genomic information for outbreak and AMR surveillance and management (Harrison et al., 2013; Besser et al., 2019; Delgado-Suárez et al., 2021). However, WGS requires culture, isolation, and nucleic acid extraction prior to sequencing.

A shotgun metagenomic sequencing approach combines the rapidness of using DNA extracted directly from clinical samples with a broad, untargeted view of all genetic information in a sample. Metagenomics has the potential to find multiple pathogens and ARGs in a single sequencing run without the need for pathogen isolation or specifying a known genetic target (Adewusi et al., 2024).

Previous proof-of-concept work using third generation Oxford Nanopore Technology (ONT) for long-read metagenomic sequencing of nasal swabs collected from feedlot cattle has shown multiple advantages compared with traditional laboratory methods (Freeman et al., 2022). Bacterial BRD pathogens, including the difficult-to-culture *Mycoplasma bovis*, were reliably identified. Not only did long-read metagenomic sequencing detect BRD pathogens more frequently than bacterial culture but it was also faster and produced some information about the presence of ARGs in the sample. However, concordance between ARGs detected by long-read metagenomic sequencing and phenotypic resistance detected by AST was limited likely due to relatively low sequence coverage of target BRD organisms and excess of bovine-derived host sequence, even with extensive host-depletion. Among 25 samples, the average proportion of non-host-derived sequence was 6%. An excessive host to non-host ratio in sequencing output reduced the ability to characterize the sample for the presence of pathogen and ARG. Analyses with short-read sequencing have shown that high ratios of host to non-host data reduced the sensitivity of microbiome and resistome profiling (Zaheer et al., 2018; Pereira-Marques et al., 2019).

Developing a reliable and cost-effective detection strategy for BRD pathogens and ARGs based on long-read metagenomic sequencing of samples requires more bacterial sequencing coverage than was previously achieved. This additional coverage could be facilitated by increasing the relative amount of non-host to host in the sequenced DNA. Our objective was to evaluate the use of a low-cost non-selective bacterial enrichment of bovine nasopharyngeal swabs as a means of increasing the proportion of target species of interest relative to host biomass. In this study we demonstrate that bacterial enrichment enhances the detection of BRD pathogens (*Pasteurella multocida*, *Mannheimia haemolytica*, and *Histophilus somni*) and species-associated ARGs, in addition to allowing us to detect *M. bovis* and *Bibersteinia trehalosi*. Our approach of using non-selective enrichment increased the proportion of sequenced on-target DNA, resulting in the reliability and utility of long-read metagenomic sequencing of nasal swab samples for diagnostics and surveillance. Additionally, using previously collected frozen swabs provided an opportunity to evaluate the potential of this method to assess archived samples.

## 2 Methods

### 2.1 Sample selection

We tested frozen swab heads from deep nasopharyngeal (DNP) swabs collected from 20 beef calves stored at −80°C as part of a larger sample collection. The samples used in this experiment were selected for a range of culture and phenotypic AST outcomes. Overall, 10 of the 20 samples were collected in 2020 from a single pen of calves sampled at 6 days on feed (DOF). Six samples were collected from calves sampled at 13 DOF from other pens from the same study, where calves arrived from October to early December 2020. Four samples were collected in 2021 from different early-, mid-, and late-filled feedlot pens (Supplementary Table S1A). The research protocols and procedures for this study were approved by the University of Saskatchewan Animal Care Committee (AUP 20190069).

### 2.2 Animals and sample collection

Samples were collected at a research feedlot operated by the University of Saskatchewan from 1,600 recently weaned mixed-breed steers purchased from a western Canadian auction market in the fall of 2020 and 2021. Calves were restrained in a hydraulic chute with a neck-extender, and three DNP swabs were collected from each calf from alternating nostrils (collection protocol details in Supplementary material). All three DNP swabs per calf were pooled in the same vial.

### 2.3 Initial sample processing

Samples were transported to the University of Saskatchewan for processing. The pooled samples (three swabs from each calf) were vortexed for 1 min to release biomass from the swab to the transport medium, and an aliquot was submitted to Prairie Diagnostic Services, Inc. (PDS; Saskatoon, Saskatchewan, Canada) for culture and AST.

### 2.4 Culture and antimicrobial susceptibility testing

In total, 10 μL inoculation loop of sample was cultured on 5% Columbia sheep blood (BA) and chocolate agar (CHOC) plates and incubated at 35°C for 18 h in an environment of 5% CO_2_ to isolate *M. haemolytica*, *P. multocida*, and *H. somni*. Bacterial colonies were incubated for 18 h and 42 h. Isolates of interest were identified using the MALDI-TOF MS Microflex LT instrument (Bruker Daltonik, Bremen, Germany) and the MALDI-TOF MS Biotyper Microflex LT Compass version 1.4 software with MSP library, according to the manufacturer’s guidelines. Isolate abundance was scored using a semi-quantitative scale (trace, 1+ to 4+) based on counts of visible colonies following streaking plates by quadrants (identification and quality control details in Supplementary material).

Susceptibility was measured by a commercially available serial broth microdilution panel using the Sensititre^™^ platform and the commercially available bovine BOPO7F Plate (ThermoFisher Scientific^™^, Thermo Fisher Scientific, Waltham, Massachusetts, United States), following the manufacturer’s instructions and recommendations for testing and quality control (antimicrobials and concentration ranges in Supplementary material). *E. coli* ATCC 25922, *Staphylococcus aureus* ATCC 29213, and *H. somni* ATCC 700025 were used to evaluate the performance. The minimum inhibitory concentration (MIC) plate was placed and read on the BIOMIC^®^ V3 microplate reader. The MIC value was considered equal to the lowest concentration of antimicrobial that inhibited visible growth.

The MICs were compared against breakpoints designated by the Clinical and Laboratory Standards Institute (CLSI) for the pathogens of interest. Isolates with MIC values considered intermediate were categorized as “susceptible” for all analyses (CLSI, 2023).

### 2.5 Sample processing for molecular and genomic testing

For samples collected in 2020, 2 mL of the remaining transport medium was centrifuged at 4,000 × g for 10 min to pellet biomass. Then, 900 μL of supernatant was decanted, and the pellet was resuspended in the remaining transport medium and host-depleted using the HostZERO™ Microbial DNA Kit (Zymo Research, Irvine, California, United States), according to the manufacturer’s recommended protocol and extracted using the QIAGEN DNEasy Blood and Tissue Vacuum Kit (QIAGEN, Hilden, Germany). The dry, swab heads were then stored in cryovials at −80°C without media or cryoprotectant.

Dry swab heads for all samples/years were removed from −80°C for enrichment. The swab head was thawed briefly at room temperature and placed aseptically into sterile glass vials with 14 mL of BHI medium containing 1% glucose and a stir bar. Glass vials were sealed with air permeable Air ‘o Top membranes (Thomson Instrument Company, Oceanside, California, United States). Cultures were grown with vigorous aeration at 35°C for 22 h. During this period, 1 mL of each sample was retrieved at 0 h and 8 h and, subsequently, every 2 h until the sample had reached the stationary phase of growth. Stationary phase was determined by optical density (OD) that indicated culture saturation at three consecutive timepoints. Each 1 mL sample was pelleted at 4,000 × g for 10 min, and the medium was discarded. Bacterial pellets were stored at −20°C overnight. Nucleic acid extraction of the resulting enriched bacterial pellets was performed using the Gentra Puregene Buccal Cell Kit (QIAGEN, Hilden, Germany), according to the manufacturer’s instructions.

DNA concentration was determined using Qubit™ 1x dsDNA Broad Range (BR) or High Sensitivity (HS) Assay Kits (Invitrogen, Carlsbad, California, United States), according to the manufacturer’s specifications. The extracted DNA was stored at 4°C for <1 month in TE-buffer until library preparation and qPCR.

### 2.6 qPCR

To identify *M. haemolytica*, *P. multocida*, and *H. somni,* qPCR was performed in triplicate on the Aria MX (Agilent Technologies, Santa Clara, California, United States) using Taqman^™^ Fast Advanced MasterMix (Invitrogen, Carlsbad, California, United States). All samples were normalized to 10 ng/μl for amplification and compared against a standard curve. Total microbial load was quantified by targeting the bacterial 16S rRNA gene (Nadkarni et al., 2002), and the abundance of organisms of interest was quantified by targeting species-specific marker genes (Kishimoto et al., 2017). The results from qPCR were examined to identify time point(s) with the highest concentrations among all BRD organisms of interest for comparative testing after the stationary phase of growth was attained.

### 2.7 Library preparation and sequencing

Two candidate optimal enrichment timepoints were identified based on qPCR results for *M. haemolytica*, *P. multocida*, and *H. somni*. DNA from the optimal timepoints and an aliquot of DNA that was extracted without enrichment were selected for further testing. Sequencing libraries were prepared using the ONT ligation kit SQK-LSK109 and native barcoding kit (EXP-NBD104 and EXP-NBD114), according to the manufacturer’s instructions, with the following reductions to reaction volumes: repair and end prep reactions were scaled to 15 μL, and barcode ligation was scaled to 20 μL. Eight barcoded samples were normalized and pooled into each library, resulting in seven sequencing runs, each run containing eight samples. Sequencing libraries were quantified with the Qubit^™^ 1x High Sensitivity (HS) Assay Kit (Invitrogen, Carlsbad, California, United States). Overall, 200 ng of each prepared library was loaded onto an FLO-MIN106 flow cell and sequenced on an ONT GridION device for 72 h.

### 2.8 Bioinformatic analysis

ONT GridION default run parameters and high-accuracy Guppy (v4.0) real-time base calling were used to process raw signal data and remove reads with an average quality < Q7, after which terminal and internal adapters in split reads were removed with Porechop v0.2.4 (Wick et al., 2017). Reads shorter than 100 bp were removed using NanoFilt v2.6.0, and sequence statistics were calculated using NanoStat v1.5.0 (De Coster et al., 2018).

Kraken2 v2.0.8-beta (Wood et al., 2019) was used to classify host and non-host reads with a custom database. The database included all complete genomes in NCBI RefSeq for the bacterial, viral, and archaeal domains on 17 October 2020, as well as the *Bos taurus* reference genome assembly ARS-UCD1.2_Btau5.0.1Y, which consists of the ARS-UCD1.2 genome assembly (Elsik et al., 2009; Rosen et al., 2020). Following classification, reads were divided into two groups using the KrakenTools v1.0 utility extract_kraken_reads.py: those assigned to the *B. taurus* genome were placed into the “host” dataset and those were classified as any other taxa or were unclassified and placed into the “non-host” dataset. Chimeric reads were retrieved from the host dataset as described by Freeman et al. (2022). Taxonomic abundance estimates for organisms of interest were computed by Bracken v2.5 (Lu et al., 2017) from the “non-host” dataset after removing host-like sequences using the KrakenTools script filter_bracken_out.py. The number of reads and total base pairs were reported for BRD bacteria, such as *M. haemolytica*, *P. multocida*, *H. somni*, *M. bovis*, and *B. trehalosi*.

Antimicrobial resistance genes were identified in non-host reads using ABRicate v.1.0.1 (Seemann, n.d.) and AMRFinderPlus v3.9.8 (Feldgarden et al., 2019), both with the NCBI Bacterial Antimicrobial Resistance Reference Gene Database (PRJNA313047, version 2020-12-17). ABRicate was also run using the Comprehensive Antimicrobial Resistance Database (CARD) (Alcock et al., 2019) and MEGARes 2.0 database (Doster et al., 2019). For AMRFinderPlus, the minimum percent identity and percent coverage thresholds were set to 60%, as it is more stringent in reporting ARGs, and the *-plus* option was used to direct the program to search for genes involved in virulence, biocide, heat, metal, and acid resistance. Default parameters were used for ABRicate (80% minimum percent identity and percent coverage).

Theoretical genome coverage was calculated as the sum of the lengths of reads classified as a particular BRD pathogen divided by the size of its reference genome (*M. haemolytica*: 2.8 Mb [NCBI GCF_002285575.1], *P. multocida*: 2.3 Mb [NCBI GCF_002073255.2], *H. somni*: 2.2 Mb [NCBI GCF_000019405.1], *M. bovis*: 0.9 Mb [NCBI GCF_001930225.1], and *B. trehalosi*: 2.3 Mb [NCBI GCF_000521725.1]).

### 2.9 Statistical analysis

Sequence statistics (number of reads, total base pairs, and theoretical genome coverage) and qPCR results for organism detection were summarized as medians. qPCR copy numbers and sequence statistics were compared between culture-positive and culture-negative samples for no enrichment and 10h and 14h enrichment using the Wilcoxon rank sum test for each organism (StataSE ver 18.0, StataCorp, College Station, TX). The Wilcoxon signed-rank test was used to compare the total base pairs detected for each organism among matched pairs of samples that were unenriched to samples enriched for 10h and 14h. The number of total base pairs was also compared with qPCR concentration for the 10h and 14h enrichment scenarios using Spearman’s correlation coefficient.

Concordance was assessed between genomic detection, defined by surpassing a threshold of sequencing reads per organism, and the traditional culture results (either positive or negative) using the kappa statistic for each organism and enrichment duration. In the present study, the cutoff representing species detection with metagenomics was estimated based on the distribution of read counts to optimize the distinction between culture positive and negative samples, leveraging previously reported metagenomic sequencing examples from the literature that used cutoffs of 100 and 1,000 reads (Zhang et al., 2022; Liu et al., 2023). Kappa was interpreted as 0.81–1.0 almost perfect agreement; 0.61–0.80 substantial agreement, 0.40–0.60 moderate agreement, 0.21–0.40 fair agreement, and 0.01–0.20 none to slight agreement (Dohoo et al., 2009).

For AMR and ARG detection for antimicrobials of interest in managing BRD, statistical analyses included kappa to assess agreement between MIC-based AST results and detection of ARGs, Wilcoxon rank sum test to assess differences in ARG numbers between isolates with and without phenotypic AMR, and the Wilcoxon singed-rank test to assess differences in ARGs among unenriched samples and samples enriched for 10h and 14 h. *p*-values ≤ 0.05 were considered significant.

## 3 Results

### 3.1 Trajectory of bacterial growth from frozen swabs

**Figure 1:**
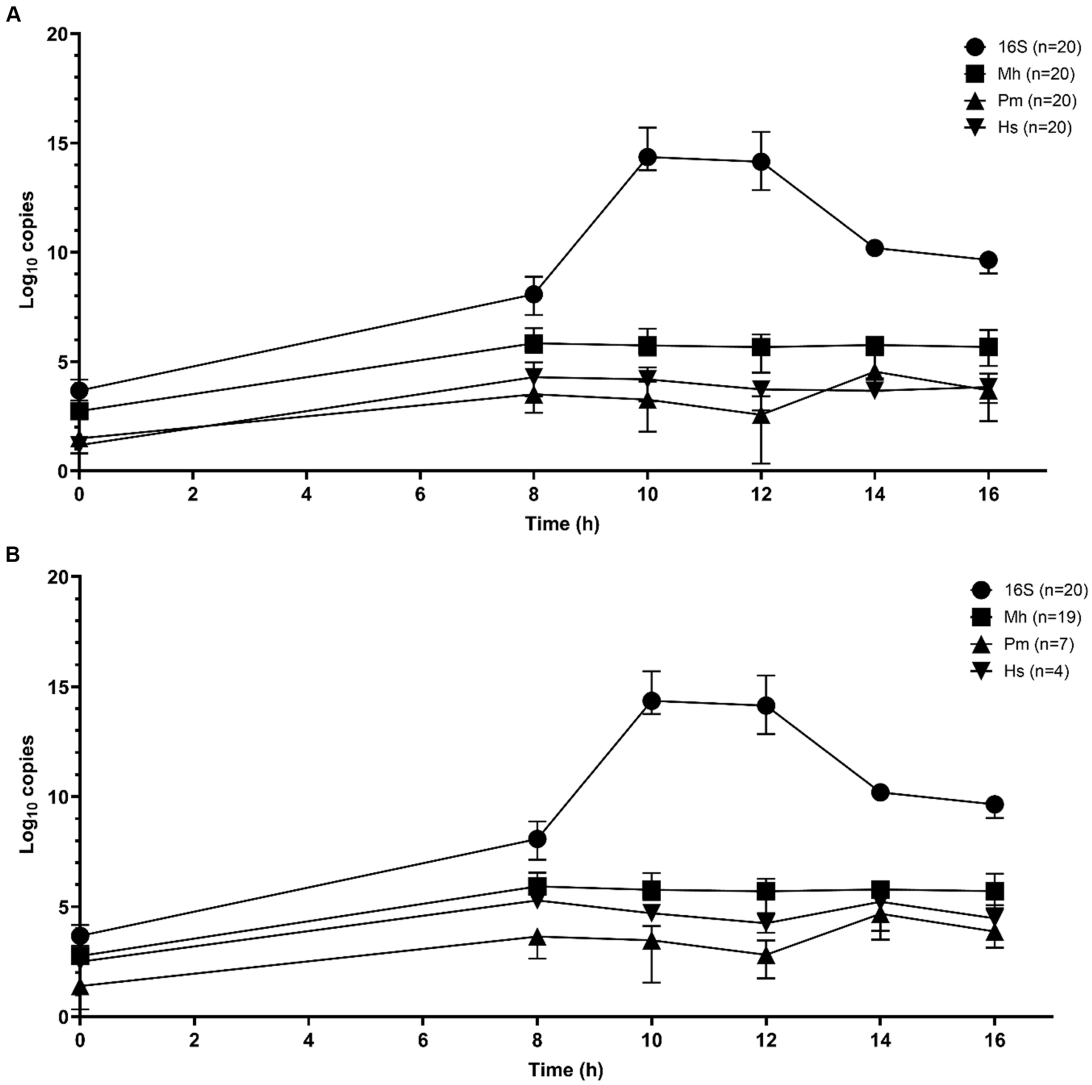
Mean qPCR copy numbers (three repeats per n samples—median value and interquartile range) of bacterial Bovine Respiratory Disease pathogens for DNA samples extracted from frozen swabs from 0 h to 16 h of O_2_ incubation in BHI broth with 1% glucose (*n* = 20 samples) for total 16S rRNA gene copies, *M. haemolytica* (Mh), *P. multocida* (Pm), and *H. somni* (Hs) (Nadkarni et al., 2002; Kishimoto et al., 2017). **(A)** All samples and **(B)** the results restricted to samples that were culture-positive for *M. haemolytica* (19 samples), *P. multocida* (7 samples), and *H. somni* (4 samples).

Sequential qPCR testing was used initially to screen samples from all time periods and select candidate enrichment time points for metagenomic sequencing. Total bacterial abundance continued to increase up to 10 h of incubation, as determined by qPCR targeting the 16S rRNA gene (Figures 1A,B). *M. haemolytica* plateaued after 8 h. *P. multocida* and *H. somni* were highest at 14 h. Samples from 10 and 14 h were selected for metagenomic sequencing; 10 h was the first time point after total bacterial abundance peaked and 14 h represented as the highest joint concentrations of *M. haemolytica, P. multocida*, and *H. somni.*

### 3.2 Sequence statistics

After removal of short (<100 bp) and low-quality (<Q7) sequences, the median total base pairs of data available for analyses, number of total reads, read lengths, and read quality were summarized for unenriched, 10- and 14-h enriched samples (Supplementary Tables S1A,B). The median read lengths were longer for enriched than unenriched samples. Non-selective enrichment increased the percentage of non-host DNA in most samples (Table 1). The percentage of non-host sequence measured by total base pairs and total non-host reads was less than 7% for all non-enriched samples. The median percentage of non-host sequence was higher for enriched samples; 54% (range 7–93%) for 10 h and 61% (range 38–94%) for 14 h (Table 1).

**Table 1:** Percentage non-host sequence based on total base pairs and reads for samples undergoing bacterial enrichment for 10h and 14h compared with DNA from samples that were not enriched.

		Subset matching no enrichment DNA (*n* = 16)			Full data set (*n* = 20)		
		Median	Minimum	Maximum	Median	Minimum	Maximum
Percent non-host sequence (based on total base pairs)	No enrichment	0.3	0.1	6	–	–	–
	10 h	54	7	93	56	7	93
	14 h	61	38	94	61	38	94
Percent non-host reads	No enrichment	0.9	0.3	7	–	–	–
	10 h	38	6	76	34	6	76
	14 h	37	17	84	36	17	84

**Figure 2:**
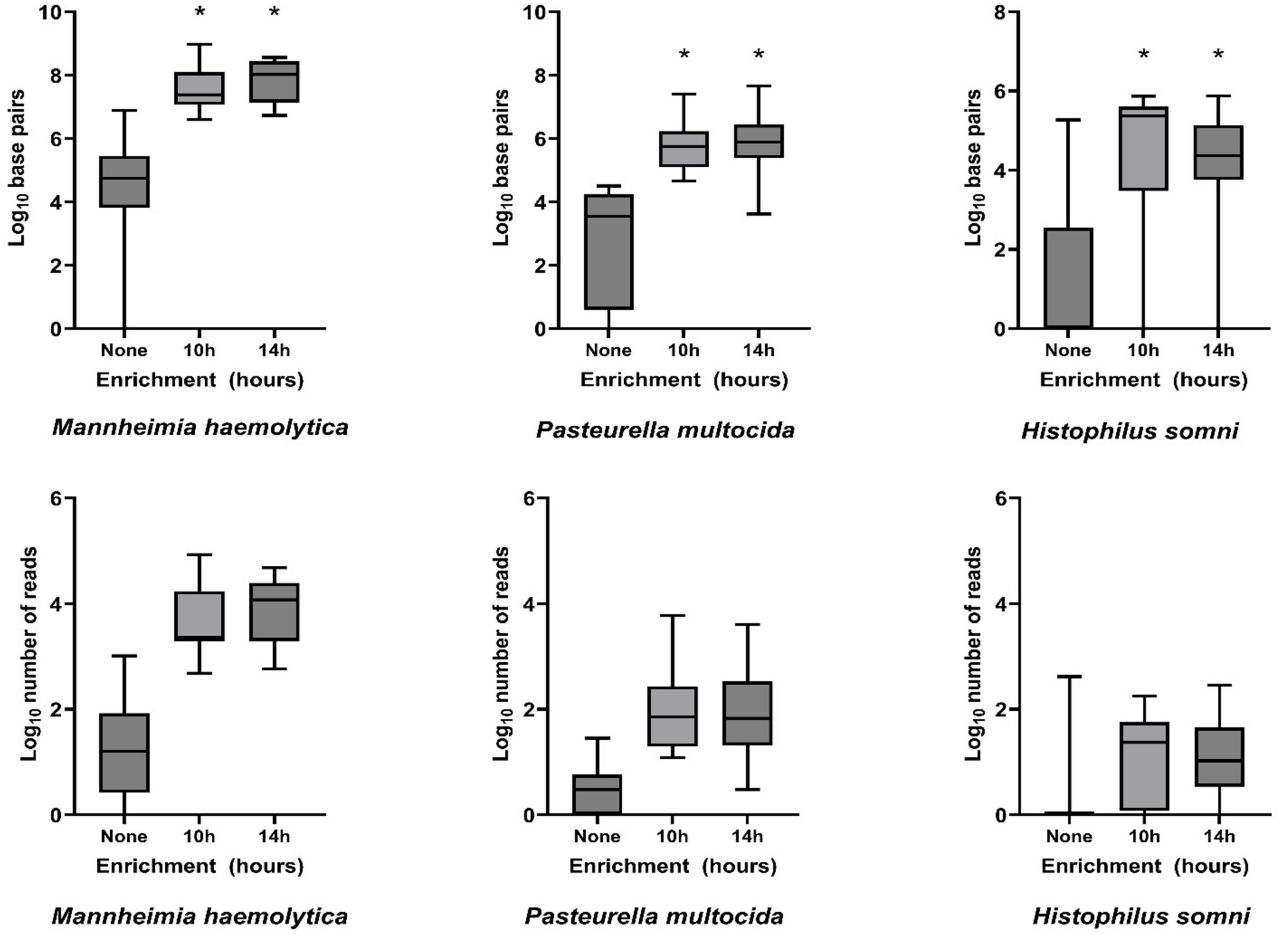
Log_10_-transformed total DNA base pairs and total number of DNA read counts for each enrichment treatment (none, 10 h, and 14 h, *n* = 16 for each) of frozen swabs to detect bacterial Bovine Respiratory Disease pathogens by metagenomic sequencing. Box and whisker plots: boxes include the median and upper and lower quartiles; whiskers include the minimum and maximum values. *p < 0.01 on Wilcoxon signed-rank test of the total base pairs for enrichment treatment compared with no enrichment.

Bacterial enrichment increased the percentage of sequence data from BRD pathogens relative to unenriched samples. The increase was two to almost four orders of magnitude higher for read numbers and total sequence length (bp) for *M. haemolytica* and two orders of magnitude for *P. multocida* (Table 2, Figure 2, Supplementary Tables S2A,B). There were no comparable culture-positive unenriched samples for *H. somni* (Supplementary Table S2C).

**Table 2:** Taxonomic classification statistics for Bovine Respiratory Disease pathogens: culture results, number of reads, amount of sequence (total base pairs—bp), and the theoretical genomic coverage of that sequence for different sample enrichment treatments.

Sample type and ID	Culture	No enrichment			10h enrichment				14h enrichment			
		Number of reads	Total base pairs	Theoretical coverage	qPCR (copies)	Number of reads	Total base pairs	Theoretical coverage	qPCR (copies)	Number of reads	Total base pairs	Theoretical coverage
***Mannheimia haemolytica***												
Median all samples	*n* = 20	16	5.6 × 10^4^	0.02	1.94 × 10^6^	2.30 × 10^3^	2.42 × 10^7^	8.6	2.93 × 10^6^	1.19 × 10^4^	1.06 × 10^8^	37.9
Median culture positives	*n* = 19	16	5.6 × 10^4^	0.02	2.38 × 10^6^	2.52 × 10^3^	2.56 × 10^7^	9.2	3.31 × 10^6^	1.26 × 10^4^	1.11 × 10^8^	39.7
Median all 2020 samples	*n* = 16	16	5.6 × 10^4^	0.02	1.94 × 10^6^	2.30 ×10^3^	2.42 × 10^7^	8.6	2.93 × 10^6^	1.19 × 10^4^	1.06 × 10^8^	37.9
Median 2020 culture positives	*n* = 16	16	5.6 × 10^4^	0.02	1.94 × 10^6^	2.30 × 10^3^	2.42 × 10^7^	8.6	2.93 × 10^6^	1.19 × 10^4^	1.06 × 10^8^	37.9
Median culture negatives	*n* = 1	Not sequenced			782	430	3.62 × 10^6^	1.3	71.4	697	5.24 × 10^6^	1.9
Kappa (reads > 1,000 vs. culture)	*n* = 20	NA			κ = 0.35 Fair agreement				κ = 0.64 Substantial agreement			
Spearman’s ρ/*p*-value (PCR ng vs. total bp)	*n* = 20				0.86/*p* < 0.001				0.87/*p* < 0.001			
WSR Test *p*-value (exact): comparison of total base pairs	*n* = 16	NE vs. 10 h*p* < 0.001	NE vs. 14 h*p* < 0.001	10h vs. 14 h*p* = 0.07								
*Pasteurella multocida*												
Median all samples	*n* = 20	3	4.22 × 10^3^	0.002	2.04 × 10^3^	72	4.89 × 10^5^	0.2	4.79 × 10^4^	68	6.05 × 10^5^	0.3
Median culture positives	*n* = 7	7	1.39 × 10^4^	0.01	1.66 × 10^3^	420	2.24 × 10^6^	1	3.04 × 10^4^	504	4.15 × 10^6^	1.8
Median all 2020 samples	*n* = 16	3	4.22 × 10^3^	0.002	1.10 × 10^3^	72	5.85 × 10^5^	0.3	3.64 × 10^4^	68	7.80 × 10^5^	0.3
Median 2020 culture positives	*n* = 6	7	1.39 × 10^4^	0.01	1.66 × 10^3^	420	2.24 × 10^6^	1	3.04 × 10^4^	504	4.15 × 10^6^	1.8
Median culture negatives	*n* = 13	1	5.62 × 10^2^	0.0002	1.10 × 10^3^	22	1.87 × 10^5^	0.1	2.25 × 10^5^	43	3.11 × 10^5^	0.1
WRS test: *p*-value (exact)	*n* = 20	*p* = 0.11	*p* = 0.23	p = 0.23	*p* < 0.001	*p* < 0.001	*p* = 0.001	p = 0.001	*p* = 0.001	*p* = 0.006	*p* = 0.005	*p* = 0.005
Kappa (reads > 100 vs. culture)	*n* = 20	NA	(κ = 0.11 - reads >1 vs. culture)		κ = 0.68 Substantial agreement				κ = 0.56 Moderate agreement			
Spearman’s ρ/*p*-value (PCR ng vs. total bp)	*n* = 20				0.77/*p* < 0.001				0.79/*p* < 0.001			
WSR Test *p*-value (exact): comparison of total base pairs	*n* = 16	NE vs. 10 h*p* < 0.001	NE vs. 14 h*p* < 0.001	10 vs. 14 h*p* = 0.03								
*Histophilus somni*												
Median all samples	*n* = 20	0	0	0	1.22 × 10^4^	42	3.50 × 10^5^	0.2	4.60 × 10^3^	17	5.83 × 10^4^	0.03
Median culture positives	*n* = 4	Not sequenced			6.81 × 10^4^	482	6.81 × 10^4^	482	5.56 × 10^6^	2.5	1.85 × 10^5^	4,298
Median all 2020 samples	*n* = 16	0	0	0	3.90 × 10^3^	15	6.37 × 10^4^	0.03	4.02 × 10^3^	14	4.33 × 10^4^	0.02
Median 2020 culture positives	*n* = 0	No culture positives: 2020 samples			No culture positives: 2020 samples				No culture positives: 2020 samples			
Median culture negatives	*n* = 16	0	0	0	3.90 × 10^3^	24	2.39 × 10^5^	0.1	3.67 × 10^3^	11	2.35 × 10^4^	0.01
WRS test *p*-value (exact)	*n* = 20	NA	NA	NA	*p* = 0.02	*p* < 0.001	*p* < 0.001	*p* < 0.001	*p* = 0.03	*p* < 0.001	*p* < 0.001	*p* < 0.001
Kappa (reads > 100 vs. culture)	*n* = 20	NA			κ = 0.63 Substantial agreement				κ = 0.74 Substantial agreement			
Spearman’s ρ/*p*-value (PCR ng vs. total bp)	*n* = 20				0.49/*p* = 0.03				0.68/*p* = 0.001			
WSR Test *p*-value (exact): comparison of total base pairs	*n* = 16	NE vs. 10 h*p* < 0.001	NE vs. 14 h*p* = 0.005	10h vs. 14 h*p* = 0.19								


WRS test: Wilcoxon Rank-Sum test of value (qPCR concentration, number of reads, total bp, or coverage) of culture-positive compared with culture-negative samples for 10h and 14h enrichment. NA: Kappa not calculated no culture positives; NE: no enrichment samples.

WSR test: Wilcoxon Signed-Rank test of read counts of comparisons between unenriched and enriched for 10h and enriched for 14h.

In the absence of enrichment, only one sample produced >1x theoretical genome coverage for any organism (Supplementary Tables S2A–C). This sample had 2.8x coverage for *M. haemolytica* (Supplementary Table S2A) and was the only sample where a culture was semi-quantitatively scored as 3+ out of a possible 4+ abundance of colony growth on the culture plate (Table 2). In contrast, at 14 h, *M. haemolytica* was detected at >30x theoretical coverage in 11 of 19 culture-positive samples (all samples >1.8x coverage) (Supplementary Table S2A). At 14 h, *P. multocida* was detected at >1.6x theoretical coverage in five of seven culture-positive samples (Supplementary Table S2B), and *H. somni* was detected at >8x theoretical coverage in all four culture-positive samples (Supplementary Table S2C). The median theoretical coverage was ≤0.1 for culture negative samples of *P. multocida* and *H. somni* at 10h and 14 h (Supplementary Tables S2B,C). There was only one sample culture negative for *M. haemolytica* with a theoretical coverage of 1.3 for 10 h and 1.9 for 14 h (Supplementary Table S2A). Notably, this sample was also qPCR positive.

*M. bovis* was only detected in enriched samples (6/20 at 10 h and 2/20 at 14 h) and *B. trehalosi* was detected in more enriched (12/20 at 10 h and 18/20 at 14 h) than unenriched samples (3/16) (*p* < 0.05) (additional details are included in Supplementary material).

### 3.3 Concordance for species detection

*M. haemolytica* had been cultured from 19 of 20 samples, followed by *P. multocida* (*n* = 7) and *H. somni* (*n* = 4). Two BRD pathogens were co-isolated from 10 of 20 samples (Supplementary Tables S2A–C).

Culture detection for each of the three organisms of interest was compared with taxonomic classification of sequencing data (Table 2). All 16 unenriched samples (Table 2, Supplementary Table S2A) were *M. haemolytica* positive, and kappa could not be calculated. One unenriched sample with a 3+ abundance culture score had >1,000 *M. haemolytica* reads and the rest had <250 reads. In total, 6 of the 16 culture-positive unenriched samples from 2020 had <10 reads for *M. haemolytica*.

As there was only one culture-negative sample, the estimation of kappa for *M. haemolytica* was limited for the 20 enriched samples. However, there was substantial agreement (κ = 0.64) between culture positivity and samples with >1,000 reads for 14h. There was very good correlation between the qPCR results and total base pairs sequenced for *M. haemolytica* at 10 h (ρ = 0.86) and 14h (ρ = 0.87) (Table 2, Supplementary Table S2A).

There were no samples with >100 *P. multocida* reads in the unenriched group (Table 2, Supplementary Table S2B). If samples were considered positive at ≥1 *P. multocida* reads, agreement of unenriched sequencing with culture results was poor (κ = 0.11). Agreement between detection of >100 reads and culture positivity was substantial (κ = 0.68) at 14 h and moderate (κ = 0.56) at 10 h (Table 2). At 14 h, two *P. multocida* culture-negative samples had >100 reads; one was strong positive for *P. multocida* on qPCR suggesting a false-negative culture (Supplementary Table S2B). At 10h and 14h, culture-positive-enriched samples had higher (*p* < 0.01) numbers of *P. multocida* reads, total base pairs, and theoretical genome coverage than culture-negative samples; whereas, there were no significant differences without enrichment (Table 2). There was also good correlation between qPCR results and total base pairs for *P. multocida* at 10h (ρ = 0.77) and 14 h (ρ = 0.79) (Table 2).

For *H. somni* (Table 2, Supplementary Table S2C), all 2020 samples were culture-negative. In the 10h- and 14h-enriched samples, the agreement between total *H. somni* reads >100 and culture-positive samples was substantial (κ = 0.63, κ = 0.74). Correlation was satisfactory to good between qPCR results and total base pairs of *H. somni* at 10 (ρ = 0.49) and 14 h (ρ = 0.68) (Table 2). The 10h- and 14h-enriched samples had higher (*p* < 0.001) numbers of *H. somni* reads, total base pairs, and theoretical genome coverage in culture-positive versus culture-negative samples (Table 2).

### 3.4 ARG detection and concordance for enriched samples

Concordance was summarized between culture of target BRD bacteria with phenotypic AST based on MICs (Supplementary Table S3) and identification of known ARGs (Supplementary Tables S4, S5) for unenriched samples, where ARGs were detected and for all samples enriched for 10 h and 14 h (Tables 3–5). The most common types of phenotypic resistance based on CLSI breakpoints were for the macrolides gamithromycin and tulathromycin, with only one tested sample with a tetracycline resistance isolate; all three antimicrobials are used in BRD management (Brault et al., 2019) (Tables 3–5, Supplementary Table S3). Samples with sulfadimethoxine MICs >256 μg/mL were also considered in the analysis based on the prevalence (Supplementary Table S3) and the use of trimethoprim-sulfamethoxazole in the treatment of BRD (Brault et al., 2019).

**Table 3:** Concordance between phenotypic antimicrobial resistance and number of reads with antimicrobial resistance genes (ARGs) within *Mannheimia haemolytica* reads.

	Antimicrobial resistance and counts of resistance genes: *Mannheimia haemolytica*											
	Macrolides				Sulfonamides				Tetracyclines (*tet(H)*)			
Sample ID	AST	Unenriched	10 h	14 h	AST	Unenriched	10 h	14 h	AST	Unenriched	10 h	14 h
2045Bi2-003	S	0	2	0	SUL	0	3	0	TET-I	0	1 (1)	1 (1)
2045Bi2-004	No MIC	0	3	0	No MIC	0	40	48	No MIC	0	41 (36)	52 (46)
2045Bi2-018	S	0	0	0	SUL	0	12	37	TET-I	0	6 (6)	38 (33)
2045Bi2-023	GAM, TUL	3	304	50	SUL	2	183	31	S	1 (0)	20 (0)	5 (0)
2045Bi2-046	No MIC	0	2	0	No MIC	0	9	4	No MIC	0	7 (7)	2 (2)
2045Bi2-053	No MIC	0	4	0	No MIC	0	4	4	No MIC	0	1 (1)	0 (0)
2045Bi2-055	No MIC	0	0	4	No MIC	0	0	2	No MIC	0	0 (0)	0 (0)
2045Bi2-063	No MIC	0	1	0	No MIC	0	1	0	No MIC	0	0 (0)	0 (0)
2045Bi2-067	GAM, TUL	0	2	59	SUL	0	3	39	S	0	0 (0)	7 (0)
2045Bi2-070	No MIC	0	0	0	No MIC	0	16	39	No MIC	0	11 (9)	18 (16)
2045Ai2-012	GAM, TUL	0	6	33	SUL	0	4	23	S	0	1 (0)	2 (0)
2046bi2-013	GAM, TUL	0	34	127	SUL	0	26	80	S	0	0 (0)	9 (0)
2045Bix2-015	GAM, TUL	0	33	23	SUL	0	20	19	S	0	4 (0)	3 (0)
2048Ai2-036	GAM, TUL	0	60	107	SUL	0	32	72	S	0	2 (0)	4 (0)
2048Ai2-083	GAM, TUL	0	0	2	SUL	0	0	7	S	0	2 (2)	4 (3)
2046Ai2-095	GAM, TUL	0	2	0	SUL	0	1	0	S	0	0 (0)	0 (0)
2148Bdev3009	No MIC	No data	0	0	No MIC	No data	0	0	No MIC	No data	3 (0)	13 (0)
2145Ax8-011	S	No data	0	0	S	No data	0	0	S	No data	0 (0)	0 (0)
2146Bdev014	S	No data	0	1	S	No data	0	1	S	No data	3 (0)	13 (0)
2146Bdev13	ND	No data	0	1	ND	No data	0	1	ND	No data	2 (2)	2 (2)
Median (R)	8/12 = 67%	0	6	42	10/12 = 83%	0	8	27	0/12 = 0%		N/T	N/T
Median (S)	4/12 = 33%	0	0	0	2/12 = 17%	N/T	0	0.5	12/12 = 100%		2 (0)	4 (0)
Median (ND)	*n* = 1	No data	0	1	*n* = 1	N/T	0	1	*n* = 1		2 (2)	2 (2)
Median (No MIC)	*n* = 7	0	1	0	*n* = 7	0	4	4	*n* = 7		3 (1)	2 (0)
Kappa (>1 ARG)	*n* = 13	N/T	κ = 0.68substantial	κ = 0.84almost perfect	*n* = 13	N/T	κ = 0.65substantial	κ = 0.65substantial	*n* = 13	N/T	N/T	N/T
WRS Test *p*-value (exact)		*p* = 0.99	*p* = 0.02	*p* = 0.01		*p* = 0.99	*p* = 0.03	*p* = 0.11		*p* = 0.99	N/T	N/T
WSR Test *p*-value (exact)	*n* = 16	NE vs 10 h*p* < 0.001	NE vs 14 h*p* = 0.008	10 vs 14 h*p* = 0.91	*n* = 16	NE vs 10 h*p* < 0.001	NE vs 14 h*p* = 0.0002	10 vs 14 h*p* = 0.14	*n* = 16	NE vs 10 h*p* = 0.50	NE vs 14 h*p* = 0.25	10 vs 14 h*p* = 0.99

AST: antimicrobial susceptibility testing result (CLSI breakpoints specific to *M. haemolytica*). GAM: gamithromycin (MIC ≥ 16 ug/ml), TUL: tulathromycin (MIC ≥ 64 ug/ml), SUL: sulfamethoxazole (MIC > 256 ug/ml) (no CLSI breakpoint), TET: tetracycline (MIC ≥ 8ug/ml), TET-I: tetracycline intermediate susceptibility (MIC = 4 ug/ml). ND: not detected (not cultured), No MIC (minimum inhibitory concentration and phenotypic susceptibility not available—excluded from concordance), S: susceptible. R: resistant. N/T: not tested (did not have ≥1 isolates with phenotypic resistance or ≥1 isolates with ARG reads). Kappa based on comparison of >1 ARG to phenotypic AMR where negative = S or ND (isolates with No MIC excluded). WRS Test: Wilcoxon Rank Sum test of read counts compared between phenotypic AMR detection where negative = S or ND. WSR Test: Wilcoxon Signed Rank test comparison of read counts between unenriched and enriched for 10h and enriched for 14h. Tetracycline resistant reads, first value is total tetracycline ARGs and value in () is specifically for *tet(H)*.

**Table 4:** Concordance between phenotypic antimicrobial resistance and number of reads with antimicrobial resistance genes (ARGs) within *Pasteurella multocida* reads.

	Antimicrobial Resistance and Counts of Resistance Genes: *Pasteurella multocida*											
	Macrolides				Sulfonamides				Tetracyclines (*tet(H)*)			
Sample ID	AST	Unenriched	10 h	14 h	AST	Unenriched	10 h	14 h	AST	Unenriched	10 h	14 h
2045Bi2-003	ND	0	0	0	ND	0	0	0	ND	0	0 (0)	0 (0)
2045Bi2-004	ND	0	0	0	ND	0	1	3	ND	0	16 (16)	16 (16)
2045Bi2-018	ND	0	0	0	ND	0	0	1	ND	0	4 (4)	10 (10)
2045Bi2-023	ND	0	20	2	ND	0	6	0	ND	0	0 (0)	0 (0)
2045Bi2-046	ND	0	0	0	ND	0	0	0	ND	0	0 (0)	3 (3)
2045Bi2-053	S	0	0	0	S	0	1	0	S	0	0 (0)	3 (1)
2045Bi2-055	ND	0	0	0	ND	0	0	0	ND	0	0 (0)	0 (0)
2045Bi2-063	ND	0	0	0	ND	0	0	0	ND	0	0 (0)	0 (0)
2045Bi2-067	ND	0	0	0	ND	0	0	0	ND	0	0 (0)	0 (0)
2045Bi2-070	ND	0	0	0	ND	0	0	0	ND	0	6 (6)	21 (21)
2045Ai2-012	ND	0	0	0	ND	0	0	0	ND	0	0 (0)	0 (0)
2046bi2-013	S	0	0	6	S	0	0	2	S	0	0 (0)	0 (0)
2045Bix2-015	S	0	2	8	SUL	0	4	3	S	0	0 (0)	1 (1)
2048Ai2-036	S	0	2	5	SUL	0	0	0	S	0	0 (0)	0 (0)
2048Ai2-083	S	0	0	0	SUL	0	8	11	S	0	2 (2)	14 (12)
2046Ai2-095	S	0	0	0	S	0	0	0	S	0	0 (0)	0 (0)
2148Bdev3009	ND	No data	0	2	ND	No data	0	0	ND	No data	0 (0)	0 (0)
2145Ax8-011	ND	No data	0	0	ND	No data	0	0	ND	No data	0 (0)	0 (0)
2146Bdev014	ND	No data	0	0	ND	No data	0	0	ND	No data	0 (0)	0 (0)
2146Bdev13	No MIC	No data	0	0	No MIC	No data	0	1	No MIC	No data	0 (0)	1 (1)
Median (R)	0/6 = 0%	N/T	N/T	N/T	3/6 = 50%	N/T	4	3	0/6 = 0%	N/T	N/T	N/T
Median (S)	6/6 = 100%	0	0	3	3/6 = 50%	N/T	0	0	6/6 = 100%	0	0 (0)	0.5 (0.5)
Median (ND)	*n* = 13	0	0	0	*n* = 13	0	0	0	*n* = 16	0	0 (0)	0 (0)
Median (No MIC)	*n* = 1	N/T	0	0	*n* = 1	N/T	0	1	*n* = 1	N/T	N/T	N/T
Kappa (>1 ARG)	*n* = 19	N/T	N/T	N/T	*n* = 19	N/T	κ = 0.60Substantial	κ = 0.48Moderate	*n* = 19	N/T	N/T	N/T
WRS Test *p*-value (exact)		N/T	N/T	N/T		N/T	*p* = 0.08	*p* = 0.08		N/T	N/T	N/T
WSR Test *p*-value (exact)	*n* = 16	NE vs 10 h*p* = 0.25	NE vs 14 h*p* = 0.13	10 vs 14 h*p* = 0.63	*n* = 16	NE vs 10 h*p* = 0.06	NE vs 14 h*p* = 0.06	10 vs 14 h*p* = 0.70	*n* = 16	NE vs 10 h*p* = 0.13	NE vs 14 h*p* = 0.02	10 vs 14 h*p* = 0.03

AST: antimicrobial susceptibility testing result (CLSI breakpoints specific to *P. multocida*). GAM: gamithromycin (MIC ≥ 16 ug/ml), TUL: tulathromycin (MIC ≥ 64 ug/ml), SUL: sulfamethoxazole (MIC > 256 ug/ml) (no CLSI breakpoint), TET: tetracycline (MIC ≥ 8 ug/ml), TET-I: tetracycline intermediate susceptibility (MIC = 4 ug/ml). ND: not detected (not cultured), No MIC (minimum inhibitory concentration and phenotypic susceptibility not available—excluded from concordance), S: susceptible. R: resistant. N/T: not tested (did not have ≥1 isolates with phenotypic resistance or ≥1 isolates with ARG reads). Kappa based on comparison of >1 ARG to phenotypic AMR where negative = S or ND (isolates with No MIC excluded). WRS Test: Wilcoxon Rank Sum test of read counts compared between phenotypic AMR detection where negative = S or ND. WSR Test: Wilcoxon Signed Rank test comparison of read counts between unenriched and enriched for 10 h and enriched for 14 h. Tetracycline resistant reads, first value is total tetracycline ARGs and value in () is specifically for *tet(H)*.

**Table 5:** Concordance between phenotypic antimicrobial resistance and number of reads with antimicrobial resistance genes (ARGs) within *Histophilus somni* reads.

	Antimicrobial resistance and counts of resistance genes: *Histophilus somni*											
	Macrolides				Sulfonamides				Tetracyclines (*tet(H)*)			
Sample ID	AST	Unenriched	10 h	14 h	AST	Unenriched	10 h	14 h	AST	Unenriched	10 h	14 h
2045Bi2-003	ND	0	0	0	ND	0	0	0	ND	1 (1)	0 (0)	0 (0)
2045Bi2-004	ND	0	0	0	ND	0	0	0	ND	0 (0)	4 (4)	1 (1)
2045Bi2-018	ND	0	0	0	ND	0	0	0	ND	0 (0)	1 (1)	1 (1)
2045Bi2-023	ND	0	0	0	ND	0	0	0	ND	0 (0)	0 (0)	0 (0)
2045Bi2-046	ND	0	0	0	ND	0	0	0	ND	0 (0)	0 (0)	0 (0)
2045Bi2-053	ND	0	0	0	ND	0	0	0	ND	0 (0)	0 (0)	0 (0)
2045Bi2-055	ND	0	0	0	ND	0	0	0	ND	0 (0)	0 (0)	0 (0)
2045Bi2-063	ND	0	0	0	ND	0	0	0	ND	0 (0)	0 (0)	0 (0)
2045Bi2-067	ND	0	0	0	ND	0	0	0	ND	0 (0)	0 (0)	0 (0)
2045Bi2-070	ND	0	0	0	ND	0	0	0	ND	0 (0)	0 (0)	2 (2)
2045Ai2-012	ND	0	0	0	ND	0	0	0	ND	0 (0)	0 (0)	0 (0)
2046bi2-013	ND	0	0	0	ND	0	0	0	ND	0 (0)	0 (0)	0 (0)
2045Bix2-015	ND	0	0	0	ND	0	0	0	ND	0 (0)	0 (0)	0 (0)
2048Ai2-036	ND	0	0	0	ND	0	0	0	ND	0 (0)	0 (0)	0 (0)
2048Ai2-083	ND	0	0	0	ND	0	0	0	ND	0 (0)	0 (0)	0 (0)
2046Ai2-095	ND	0	0	0	ND	0	0	0	ND	0 (0)	0 (0)	0 (0)
2148Bdev3009	S	No data	0	0	SUL	No data	0	0	S	No data	0 (0)	0 (0)
2145Ax8-011	S	No data	0	0	SUL	No data	0	0	S	No data	0 (0)	0 (0)
2146Bdev014	S	No data	0	0	SUL	No data	0	0	S	No data	0 (0)	0 (0)
2146Bdev13	S	No data	0	0	SUL	No data	0	0	TET	No data	4 (4)	5 (5)
Median (R)	0/4 = 0%	N/T	N/T	N/T	4/4 = 100%	N/T	0	0	1/4 = 25%	N/T	4 (4)	5 (5)
Median (S)	4/4 = 100%	N/T	0	0	0/4 = 0%	N/T	N/T	N/T	3/4 = 75%	N/T	0 (0)	0 (0)
Median (ND)	*n* = 16	0	0	0	*n* = 16	0	0	0	*n* = 16	0 (0)	0 (0)	0 (0)
Median (No MIC)	*n* = 0	N/T	N/T	N/T	*n* = 0	N/T	N/T	N/T	*n* = 0	N/T	N/T	N/T
Kappa (>1ARG)	*n* = 20	N/T	N/T	N/T	*n* = 20	N/T	N/T	N/T	*n* = 20	N/T	κ = 0.65Substantial	κ = 0.65Substantial
WRS Test *p*-value (exact)		N/T	N/T	N/T		N/T	*p* = 0.99	*p* = 0.99		N/T	0.20	0.10
WSR Test *p*-value (exact)	*n* = 16	NE vs 10 h*p* = 0.99	NE vs 14 h*p* = 0.99	10 vs 14 h*p* = 0.99	*n* = 16	NE vs 10 h*p* = 0.99	NE vs 14 h*p* = 0.99	10 vs 14 h*p* = 0.99	*n* = 16	NE vs 10 h*p* = 0.75	NE vs 14 h*p* = 0.50	10 vs 14 h*p* = 0.99

AST: antimicrobial susceptibility testing result (CLSI breakpoints specific to *H. somni*). GAM: gamithromycin (MIC ≥ 16 ug/ml), TUL: tulathromycin (MIC ≥ 64 ug/ml), SUL: sulfamethoxazole (MIC > 256 ug/ml) (no CLSI breakpoint), TET: tetracycline (MIC ≥ 8 ug/ml), TET-I: tetracycline intermediate susceptibility (MIC = 4 ug/ml). ND: not detected (not cultured), No MIC (minimum inhibitory concentration and phenotypic susceptibility not available—excluded from concordance), S: susceptible. R: resistant. N/T: not tested (did not have ≥1 isolates with phenotypic resistance or ≥1 isolates with ARG reads). Kappa based on comparison of >1 ARG to phenotypic AMR where negative = S or ND (isolates with No MIC excluded). WRS Test: Wilcoxon Rank Sum test of read counts compared between phenotypic AMR detection where negative = S or ND. WSR Test: Wilcoxon Signed Rank test comparison of read counts between unenriched and enriched for 10 h and enriched for 14 h. Tetracycline resistant reads, first value is total tetracycline ARGs and value in () is specifically for *tet(H)*.

### 3.5 Antimicrobial resistance genes in unenriched samples

Detection of ARGs in unenriched samples was limited to ≤3 reads in two samples (Tables 3–5). In one sample, where the unenriched theoretical coverage of *M. haemolytica* was 2.8 (Supplementary Table S2A, sample 2045Bi2-023), and AST phenotypes included macrolide resistance and sulfadimethoxine MICs >256 μg/mL, 2 *sul2*, 2 *mphE*, 1 *msrE*, and 1 *tet(34)* genes were detected in the unenriched sequence data (Table 3, Supplementary Tables S4, S5A). In the second sample (2045Bi2-003), which was culture-positive for *H. somni* (Supplementary Table S2C) with no detected phenotypic resistance (Table 5), a single *tet(H)* gene was identified in the unenriched sequence data (Table 5, Supplementary Tables S4, S5C).

Resistance genes were detected more frequently in enriched samples than in unenriched samples, particularly where phenotypic resistance was most prevalent (Tables 3–5). The differences between unenriched and enriched samples were significant for macrolide and sulfonamide resistance genes within *M. haemolytica* reads (Table 3) and for tetracycline resistance genes within *P. multocida* reads (Table 4). Furthermore, ARGs were more likely to be detected (> 1 ARG) regardless of enrichment status with increased total number of base pairs for *M. haemolytica* (*p* < 0.001), *P. multocida* (*p* < 0.001), or *H. somni* (*p* = 0.03).

### 3.6 Detection of ARGs coding for macrolide resistance from enriched samples

Macrolide ARGs were identified within at least two *M. haemolytica* reads (Table 3, Supplementary Tables S4, S5A) at 10h (11/20 samples) and 14 h (8/20 samples). Agreement between detection of at least two *M. haemolytica* reads with macrolide ARGs with phenotypic AMR for gamithromycin and tulathromycin was substantial (κ = 0.68) for 10 h and almost perfect (κ = 0.83) for 14 h (Table 3). The number of *M. haemolytica* reads with macrolide ARGs was also higher at 10h (*p* = 0.02) and 14 h (*p* = 0.01) in samples where *M. haemolytica* isolates displayed phenotypic macrolide resistance compared with samples susceptible *M. haemolytica* isolates.

Genes coding for macrolide resistance were identified in 463 unique *M. haemolytica* reads sequenced in the enriched samples by at least one of the NCBI, CARD, or MEGARes databases (Supplementary Tables S4, S5A). The 859 macrolide genes on *M. haemolytica* reads had a median identity of 96% (IQR, 93–97%) and a median coverage of 98% (IQR, 97–99%) (Supplemental Table S5A). The macrolide resistance-associated genes identified on *M. haemolytica* reads in these samples were *mphE* (53%, 457/859) and *msrE* (47%, 402/859). Both *mphE* and *msrE* were identified on 81% of the 463 reads that contained at least one of these genes. Both genes were also identified on *M. haemolytica* reads in all eight samples with macrolide-resistant isolates and five of the remaining eight samples with susceptible isolates.

Macrolide ARGs were also identified within at least two *P. multocida* reads (Table 4, Supplementary Tables S4, S5B) from samples at 10h (3/20 samples) and 14 h (5/20 samples). However, there were no samples with cultured macrolide-resistant *P. multocida* (Table 4, Supplementary Table S3). Macrolide ARGs (53% *msrE* (25/47) and 47% *mphE* (22/47)) were identified on 24 *P. multocida* reads in enriched samples by at least one database. The median gene identity was 96% (IQR, 89–99%) and the median coverage was 98% (IQR, 95–100%) (Supplementary Table S5B).

No macrolide resistance-associated genes were identified within the *H. somni* reads from the 20 enriched samples (Table 5, Supplementary Tables S4, S5C). None of the samples had *H. somni* isolates that displayed phenotypic resistance to macrolides (Table 5, Supplementary Table S3).

Macrolide ARGs (*msrE*, *mphE*) were identified in 46 *B. trehalosi* reads from four samples enriched for 10 h and the same four samples plus two more enriched for 14 h. Both genes had been identified in *M. haemolytica* reads from the same six samples.

### 3.7 Detection of ARGs coding for sulfonamide resistance for enriched samples

*M. haemolytica* reads containing sulfonamide ARGs (Table 3, Supplementary Tables S4, S5A) were identified at least twice in samples for 10 h (12/20 samples) and 14 h (13/20 samples). Agreement for detection of at least two *M. haemolytica* reads containing sulfonamide ARGs (*sul2* gene) and samples culture-positive for *M. haemolytica* with MIC >256 μg/mL for sulfadimethoxine were substantial (κ = 0.65) for 10 and 14 h (Table 3, Supplementary Table S3). The number of *sul2* genes detected in *M. haemolytica* reads was higher for samples with *M. haemolytica* isolates with sulfadimethoxine for MICs >256 μg/mL than those without sulfadimethoxine in 10 h-enriched samples (*p* = 0.03) but not in 14-h-enriched samples (*p* = 0.11). The *sul2* gene (*n* = 761) was identified in 735 *M. haemolytica* reads from enriched samples. The median *sul2* gene identity was 95% (IQR, 92–97%) and the median coverage was 98% (IQR, 97–99%).

Sulfonamide ARGs within *P. multocida* reads (Table 4, Supplementary Tables S4, S5B) were identified in at least two reads for samples for 10h (3/20) and 14 h (4/20). Agreement between detection of *sul2* genes and phenotypic MICs >256 μg/mL was moderate for 10h- (κ = 0.60) and 14h-enriched samples (κ = 0.48) (Table 4). There was no significant difference in the number of *sul2* genes detected for samples with *P. multocida* isolates with MICs for sulfonamides >256 μg/mL and those without MICs (Table 4). Forty *sul2* genes were identified in 28 *P. multocida* reads from enriched samples; median identity was 95% (IQR, 93–97%) and median coverage was 98% (IQR, 97–99%).

No sulfonamide ARGs were detected within *H. somni* sequence data from samples enriched for either 10h or 14 h (Table 5, Supplementary Tables S4, S5C). However, the sulfadimethoxine MIC for all four *H. somni* isolates was >256 μg/mL.

A sulfonamide ARG (*sul2*) was identified within *B. trehalosi* in 56 reads from four samples enriched for 10 h and the same four samples plus five additional samples enriched for 14 h.

### 3.8 Detection of ARGs coding for tetracycline resistance for enriched samples

Tetracycline ARGs were detected within at least two *M. haemolytica* reads (Table 3, Supplementary Tables S4, S5A) in 10h (11/20) and 14h-enriched (14/20) samples; however, there was no detected phenotypic resistance in culture-positive isolates (Table 3, Supplementary Table S3). In all enriched samples, 277 tetracycline ARGs were detected in 275 *M. haemolytica* reads. The primary tetracycline ARGs included *tet(H)* (60%, 167/277) and *tet(34)* (39%, 109/277). The median identity for the *tet(H)* genes was 95% (IQR, 93–97%) and median coverage was 98% (IQR, 97–99%) but median identity for the *tet(34)* genes was only 62% (IQR, 61–62%) and median coverage was 77% (IQR, 67–84%). The *tet(34)* genes were not detected by the Abricate option and MEGARes databases likely due to the higher cutoff of 80% identity. The *tet(H)* genes were detected in the same six samples for 10h and 14 h.

Tetracycline ARGs were detected within at least two *P. multocida* reads (Supplementary Tables S4, S5B) for 10h (4/20) and 14 h (6/20) (Table 4). The *tet(H)* gene was detected in at least two *P. multocida* reads in four samples for 10 h and five samples for 14 h. One sample contained two *tet(34)* genes with low identity scores (62%) as reported for the same gene from *M. haemolytica*. No *P. multocida* isolates were phenotypically resistant to tetracycline (Table 4, Supplementary Table S3).

The *tet(H)* genes were detected in at least two *H. somni* reads for two samples for 10h and 14 h. The *tet(H)* gene was identified in one of four *H. somni* culture-positive samples with tetracycline-resistant isolates (Table 5, Supplementary Tables S3, S4, S5C).

The tetracycline resistance gene (*tet(H)*) was identified within *B. trehalosi* in eight reads from one sample for 10 h and the same sample enriched for 14 h.

### 3.9 Other resistance genes

The only other ARGs reported at least twice in enriched samples conferred aminoglycoside resistance. The most common was *APH(3′)*. There were 282 *APH(3′)-Ia* genes identified on 278 *M. haemolytica* reads from 14 samples and 13 genes on 12 *P. multocida* reads from 4 samples (Supplementary Tables S4A,B, S5A,B). There were also 232 *APH(3′)-Ib* genes identified on 230 *M. haemolytica* reads from 14 samples, and 20 genes were identified on 18 *P. multocida* reads from eight samples.

Another identified aminoglycoside resistance gene was *APH(6)-Id*, with 245 *APH(6)-Id* genes identified on 240 *M. haemolytica* reads from 14 samples and 13 genes on 12 *P. multocida* reads from 4 samples (Supplementary Tables S4A,B, S5A,B).

No aminoglycoside ARGs were identified in *H. somni* reads (Supplementary Tables S4A,B, S5C) or *B. trehalosi* reads.

### 3.10 Detection of multiple ARGs on single reads

Multiple ARGs were detected on 739 long individual reads for 31 metagenomic analyses of 19 unique samples (Supplementary Table S6). In total, 2 of the 739 reads with multiple ARGs were from unenriched samples.

The median number of unique ARGs on each read was three (5th percentile = two, 95th percentile = four). The median length of individual reads on which multiple ARGs were found was 21,941 bp (5th percentile 7,971, 95th percentile 53,678 bp). Most reads with multiple ARGs were *M. haemolytica* (*n* = 694), with smaller numbers of *P. multocida* (n = 42) and only one *H. somni* read. The most common pattern of multiple ARGs per read was *msrE*, *mphE*, and *sul2* in 349 reads followed by *APH(3″)-Ib*, *APH(6)-Id*, *APH(3′)-Ia*, and *sul2* in 188 reads. Both macrolide ARGs and sulfonamide ARGs were detected in 45% of the 774 *M. haemolytica* reads with at least one of these genes and in at least one *M. haemolytica* read from 78% (14/18) of samples with at least one of these genes.

## 4 Discussion

Non-selective bacterial enrichment increased the amount of on-target data available from metagenomic sequencing of nasal samples from feedlot cattle enabling previously unreported robust detection of ARGs on species-specific respiratory bacterial reads in complex respiratory samples from healthy animals. Incubating the sample in growth medium for 10h and 14 h increased bacterial numbers for three important BRD pathogens of interest relative to unenriched samples and for *B. trehalosi*. In the case of *M. haemolytica* and *B. trehalosi*, enrichment also increased the read detections of ARGs for macrolide and sulfonamide resistance, and tetracycline ARGs for *P. multocida*.

Host DNA is a major impediment in metagenome analysis, particularly in nasal swab samples, where >90% of sequencing reads can be host-derived (Marotz et al., 2018; Chen and Xu, 2023; Ring et al., 2023). High proportions of host DNA reduce the sensitivity of metagenomic sequencing, especially for detecting low-abundance bacteria (Pereira-Marques et al., 2019). In this study, all 20 enriched samples resulted in >93% non-host DNA for both the 10h and 14h protocols. For the three BRD bacterial pathogens of most interest, *M. haemolytica*, *P. multocida,* and *H. somni*, non-selective enrichment increased the detection of targeted sequence by several orders of magnitude, particularly in culture-positive samples. Increased coverage of the pathogen genomes was directly associated with the detections of ARGs within identified bacterial reads for antimicrobials of interest such as macrolides and tetracyclines. Another recent study of metagenomic identification of pathogens in blood cultures also described increased ARG detection with increased total base pairs reported for target organisms of interest (Liu et al., 2023), confirming the clinical relevance of additional on-target bacterial sequence data.

At least some reads for all three BRD pathogens were detected in sequence data from samples with negative culture results, particularly for enriched samples. This finding could partially be explained by the ability of sequence-based approaches to detect DNA from growth-inhibited or dead pathogens (Shao et al., 2022). This discrepancy could also be due to the inherent sampling bias of streak plate-based identification of pathogens, wherein only 10 μL loop of the original sample is used for analysis, which could miss low-abundance organisms. In previous studies comparing detection of BRD pathogens, sequencing demonstrated positive results more frequently than culture, and the concordance between these techniques varied by organism (Bell et al., 2014; Freeman et al., 2022). However, in the present study, the sequence and qPCR data generated for enriched culture-positive samples were significantly higher than culture-negative samples.

In the present study, one advantage of metagenomic sequencing for BRD pathogen detection is that it can provide a snapshot of the nasal bacteria beyond the detection of specific organisms targeted by routine culture, such as *M. bovis* and *B. trehalosi*. However, non-selective bacterial enrichment can modify the relative quantities of identified organisms, as the community structure can be altered by variation in replication rate and overgrowth of some organisms (Jarvis et al., 2015; Leonard et al., 2015). In the present study, differences in the absolute read numbers and relative depth of sequence data were recognized for target organisms of interest. There was a higher chance of detecting ARGs of interest in samples that had higher reads and more base pairs for BRD organisms. However, the protocol was optimized to include 14h enrichment based on qPCR detection of the three primary organisms of interest. The long-read metagenomic sequencing of our samples had good agreement with culture and reflected what we expected to observe in calves when they arrive at the feedlot; initial recovery of *M. haemolytica* and *P. multocida* with increasing frequency of *H. somni* and *M. bovis* in the feeding period (Alhamami et al., 2021; Andrés-Lasheras et al., 2021; Younes et al., 2022).

While the impact of non-selective enrichment on description of the microbiome requires further research, this study demonstrates that this approach was successful in identifying primary bacteria of interest for managing BRD and enhancing detection of ARGs in these organisms. While not all organisms were expected to benefit equally from the enrichment protocol, culture times were relatively short; DNA from unculturable (i.e., dead) or difficult to culture bacteria was unlikely to be lost with enrichment, and therefore, the risk of losing taxonomic breadth was minimal (Lennon et al., 2018; Shao et al., 2022). Even *M. bovis*, which replicates slowly and requires additional nutrients not provided by the enrichment medium used in this study (McVey et al., 2013), was detected following bacterial enrichment. Furthermore, *M. bovis* was identified in calves sampled early in the feeding period when we did not expect a high prevalence (Freeman et al., 2022). However, the resulting sequencing data were limited with 0.23 as the highest observed theoretical coverage of *M. bovis* in a single sample.

In a proof-of-concept study (Freeman et al., 2022), metagenomic sequencing without bacterial enrichment detected BRD pathogens of interest more frequently than did culture, but ARGs from relevant taxa were not reliably detected due to high levels of host DNA. Our findings show that with enrichment and the resulting increased sequencing coverage of target organisms, hundreds of ARGs were detected with taxonomic resolution to the species level, with moderate and better concordance with phenotypic resistance.

Samples with *M. haemolytica* isolates displaying phenotypic resistance to the 15-member macrolides, tulathromycin and gamithromycin, were selected for this pilot study. Agreement between AST results for macrolides in *M. haemolytica* and the detection of ARGs on *M. haemolytica* reads was in agreement with 14h enrichment. The genes *mphE* and *msrE* encode a macrolide phosphotransferase protein and an ABC transporter protein, respectively (Kadlec et al., 2011), and have been consistently associated with macrolide resistance in *Pasteurellaceae* isolates derived from feedlot cattle (Alhamami et al., 2021; Andrés-Lasheras et al., 2021; Younes et al., 2022). These two genes were recovered together on 81% of individual *M. haemolytica* reads with any macrolide resistance and all of the samples with phenotypic macrolide-resistant *M. haemolytica.* The colocation of these two genes was expected as was found in previous reports using PCR and assemblies based on whole genome sequencing (Clawson et al., 2016; Snyder et al., 2019; Klima et al., 2020; Stanford et al., 2020) but has not been previously described on individual raw reads from metagenomic data.

The *erm42* gene is often present in isolates exhibiting resistance to gamithromycin, tulathromycin, and tilmicosin (Desmolaize et al., 2011; Rose et al., 2012; Snyder et al., 2019) but was not detected in these samples. Although macrolide resistance can emerge from point mutations in the 23S rRNA gene (Olsen et al., 2015); the phenotypic resistance in this study was explained by the presence of previously characterized macrolide ARGs. Macrolide ARGs were also detected on reads of BRD organisms where the specific organisms were absent on culture or AST reported for the tested isolate from the sample was susceptible to macrolides. However, smaller number of macrolide ARGs were present in the absence of phenotypic macrolide-resistant *M. haemolytica*. Metagenomics could have detected ARG reads in samples with isolates that were either not detected or not selected for MIC testing.

In this study, high MICs for sulfadimethoxine (MIC >256 μg/mL) were observed for isolates of all three BRD pathogens. Agreement between the *sul2* gene and high sulfadimethoxine MICs was at least moderate for *M. haemolytica* and *P. multocida*. However, in the four samples where sulfadimethoxine-resistant *H. somni* isolates were recovered, no *sul2* genes were detected suggesting another potential, undetected resistance mechanism. Furthermore, almost half of all *M. haemolytica* reads and four out of five samples with either macrolide or sulfonamide ARGs had both macrolide and sulfonamide ARGs. This suggests that these genes are frequently collocated potentially on integrative and conjugative elements (ICE) (Clawson et al., 2016; Beker et al., 2018). Colocation of macrolide and sulfonamide ARGs was also observed in *B. trehalosi* reads.

In this study, tetracycline phenotypic resistance was only reported in one *H. somni* isolate. The tetracycline resistance gene *tet(H)* was identified in this sample and encoded a tetracycline efflux protein. Other studies have found variable concordance with phenotypic resistance to tetracycline and *tet* genes (Owen et al., 2017; Snyder et al., 2020). The *tet(34)* gene was also identified in several samples; however, given the lack of concordance with phenotypic resistance and the low percentage identity of the *tet(34)* genes, these results were unlikely to be clinically relevant.

Concordance between genotypic ARG detection and phenotypic AST varied, and there were sufficient samples with phenotype positive and negative isolates to generate reliable metrics. However, most calculations suggested substantial agreement and significant differences in read numbers between samples with AST-positive and AST-negative isolates. Concordance can vary depending on the organism and antimicrobial, level of transcriptional expression, and sequence quality. In some previous reports, phenotypic resistance was highly correlated with known resistance determinants. Concordance between AST and WGS for *M. haemolytica* from 20 stocker calves calculated from raw data was very good for tilmicosin (κ = 0.96), tulathromycin (κ = 0.96), and tetracycline (κ = 1.0) (Snyder et al., 2020). Agreement was lower between oxytetracycline resistance and detection of *tet(H)* (κ = 0.66) and sulfonamide resistance and detection of *sul2* (κ = 0.38) for 64 WGSs for *M. haemolytica, P. multocida,* and *H. somni* isolates from beef and dairy calves (Owen et al., 2017).

This study demonstrated the use of long-read metagenomic sequencing for the detection of ARGs linked directly to bacterial species without the need for genome assembly. The detection of ARGs has been reported from long-read metagenomic sequencing for one study of aspirates from human ventilator-associated pneumonia (Chen et al., 2023) but more typically from samples with substantially less host DNA, such as those recovered from positive blood cultures (Taxt et al., 2020; Liu et al., 2023), bile cultures (Whittle et al., 2022), milk cultures (Ahmadi et al., 2023), and urine cultures (Zhang et al., 2022; Ring et al., 2023).

There were no previous reports of ARG detection within identified pathogen reads using long-read metagenomic sequencing in human or veterinary upper respiratory tract samples (Chen and Xu, 2023; Adewusi et al., 2024) with the exception of the limited success in earlier study published by our group (Freeman et al., 2022). Resistance genes were not recovered in a veterinary study of skin infections in dogs due to the high percentage of host DNA (Ring et al., 2023). None of the metagenomic studies reporting ARGs explicitly reported either individual or multiple ARGs on single long reads of the organisms of interest at the species level as described in the present study, potentially increasing the clinical relevance of the ARG detection.

Short-read data can be used for read-based detection of ARGs; however, it typically requires higher genome coverage and more computing resources compared with long-read detection (Gupta et al., 2020), making long-read metagenomic options such as our method attractive as genomics moves toward rapid diagnostic solutions to inform antimicrobial stewardship. Long-read methods such as ONT also offer access to taxonomic real-time data during the sequencing run, which may also speed up the time for results and is not currently available for short-read sequencing methods (Votintseva et al., 2017; Charalampous et al., 2019; Chan et al., 2020; Ring et al., 2023).

Although bacterial enrichment of samples has improved the sensitivity of both taxonomic and ARG detection, this method is not without drawbacks. Indeed, it adds time and complexity to sample preparation for sequencing, and it does not offer the same unbiased insight into the microbial community structure for those whose objective might be traditional microbiome research as does metagenomic sequencing of unenriched samples. However, in our case, the goal was to detect reads of specific BRD pathogens with ARGs to inform clinical decisions and not to produce a general description of the nasal microbiome. Larger studies including more animals will further validate this tool against alternative testing methods and assess whether this method might be a cost-effective option to help inform antimicrobial stewardship.

## 5 Conclusion

Long-read metagenomic sequencing of enriched DNP samples from feedlot cattle to detect BRD pathogens and ARGs shows promise as a diagnostic testing strategy for feedlot cattle production. Agreement between pathogen detection and traditional culture-based methods was improved by the enrichment step. While this step adds time and makes the process less portable, the relative benefits of improving sequence quantity for non-host reads and ARG detection outweighed these costs. This method provides additional promise for the characterization of species not specifically targeted by routine culture and susceptibility protocols with no additional diagnostic costs.

## Data availability statement

The data presented in this study were deposited to the Sequence Read Archive as submission SUB14263813 and as BioProject PRJNA1096931.

## Ethics statement

The animal study was approved by University of Saskatchewan Animal Care Committee (AUP 20190069). The study was conducted in accordance with the local legislation and institutional requirements.

## Author contributions

EH: Methodology, Writing – original draft, Software. SL: Methodology, Conceptualization, Project administration, Writing – review & editing. CF: Methodology, Writing – original draft. SO: Funding acquisition, Writing – review & editing. EM: Writing – review & editing, Data curation, Software. ML: Data curation, Software, Formal analysis, Writing – review & editing. PS: Software, Writing – review & editing. CW: Writing – review & editing, Conceptualization, Data curation, Formal analysis, Funding acquisition, Methodology, Project administration, Supervision, Validation, Visualization, Writing – original draft.
