# Supplementary material for: Bacterial enrichment prior to third-generation metagenomic sequencing improves detection of BRD pathogens and genetic determinants of antimicrobial resistance in feedlot cattle
Source: Front Microbiol. 2024 May 8;15:1386319. doi: 10.3389/fmicb.2024.1386319 (PMC11110911; doi:10.3389/fmicb.2024.1386319)
Supplement: Supplementary file 1 [file Data_Sheet_1.pdf]

Supplemental methods and results for:

Bacterial enrichment prior to third-generation metagenomic sequencing improves detection of BRD pathogens and genetic determinants of antimicrobial resistance in feedlot cattle

Emily K. Herman[1], Stacey R. Lacoste[2], Claire N. Freeman[2], Simon J. G. Otto[3,4], E. Luke McCarthy[5], Matthew G. Links[5,6], Paul Stothard[1], Cheryl Waldner[2*]

[1] Department of Agricultural, Food, and Nutritional Science, Faculty of Agricultural, Life, and Environmental Sciences, 2-31 General Services Building, University of Alberta, Edmonton, AB T6G 2H1

[2] Department of Large Animal Clinical Sciences, Western College of Veterinary Medicine, University of Saskatchewan, 52 Campus Drive, Saskatoon, Saskatchewan S7N 5B4

[3] HEAT-AMR (Human-Environment-Animal Transdisciplinary AMR) Research Group, School of Public Health, University of Alberta, 11322 89 Ave NW, Edmonton, AB, T6G 2G7

[4] Healthy Environments Thematic Area Lead, Centre for Healthy Communities, School of Public Health, University of Alberta

[5] Department of Animal and Poultry Science, College of Agriculture and Bioresources, University of Saskatchewan, 51 Campus Drive, Saskatoon, Saskatchewan S7N 5A8

[6] Department of Computer Science, College of Arts and Science, University of Saskatchewan, 110 Science Place, Saskatoon, Saskatchewan S7N 5C9

2. Methods (Supplemental details)

2.2 Sample collection protocol – additional details

The external nares were wiped with a single-use paper towel, and a double-guarded culture swab (Continental Plastic Corp., Delevan, Wisconsin, USA) was directed into the ventral meatus of the nostril. The polyester-tipped swab was advanced through the inner sheath and vigorously rotated 5-6 times against the pharyngeal mucosa. The swab was withdrawn into the inner sheath and outer guard before removal from the nostril, and 3 cm of each swab tip was cut and placed in a vial with 3 mL of liquid Amies transport medium. Two additional samples were obtained from alternating nostrils.

2.4a Culture – organism identification and quality control – additional details

When visible characteristics suggested the presence of multiple isolates of interest from one sample, representative colonies of each unique colony morphology were selected for identification. Positive and negative controls were processed for each day of sample setup and for each new media lot using *Staphylococcus aureus* ATCC 29213, *Escherichia coli* ATCC 25922, and *H. somni* ATCC 700025. Only MALDI-TOF identification scores of ≥2 and indicating secure species-level identification were used for further analysis. A plain matrix spot was run with every MALDI-TOF run to ensure no contamination.

Additionally, the same colony of interest was inoculated into Todd Hewitt broth. Isolates were then streaked from the inoculated Todd Hewitt broth onto plates specific to each bacterial type to assess purity, BA for *M. haemolytica* and *P. multocida*, and CHOC for *H. somni*.

2.4b Antimicrobial Susceptibility Testing – range of tested concentrations – additional details

Sensititre plates included doubling dilutions across the specified concentration ranges (ug/ml) to evaluate MICs for each antimicrobial (ampicillin (0.25-16 μg/ml), ceftiofur (0.25-8 μg/ml), clindamycin (0.25-16 μg/ml), danofloxacin (0.125-1 μg/ml), enrofloxacin (0.125-2 μg/ml), florfenicol (0.25-8 μg/ml), gamithromycin (1-8 μg/ml), gentamycin (1-16 μg/ml), neomycin (4-32 μg/ml), penicillin (0.125-8 μg/ml), spectinomycin (8-64 μg/ml), sulphadimethoxime (256 μg/ml), tetracycline (0.5-8 μg/ml), tiamulin (0.5-32 μg/ml), tildipirosin (1-16 μg/ml), tilmicosin (2-16 μg/ml), trimethoprim/sulfamethoxazole (2 μg/ml), tulathromycin (8-64 μg/ml), tylosin tartrate (0.5-32 μg/ml)).

3. Results (Supplemental data)

3.2 Sequence statistics – M. bovis and B. trehalosi – additional data

M. bovis was not identified by metagenomic sequencing in any of the 16 unenriched samples. In 20 samples enriched for 10 hours, *M. bovis* was identified in six samples (one read – four samples, two reads – one sample, seven reads – one sample). At 14 hours, *M. bovis* was identified in two samples; one with two reads and one with 41 reads, where 209 kbp was assigned to *M. bovis* (theoretical coverage, 0.23). At 10 hours enrichment this same sample produced 84 kbp *M. bovis* sequence (theoretical coverage, 0.09).

B. trehalosi was detected in three of 16 unenriched samples by one, two, and four reads. After 10 hours, between 1 and 24 *B. trehalosi* reads were detected in 12 of 20 samples (median, 1 read). After 14 hours, it was identified in 18 samples with a maximum of 315 reads (median, 6 reads). Culture data were not available for *M. bovis* or *B. trehalosi* in this study. There were no significant differences among different *M. bovis* sample preparation protocols ($p > 0.13$). Detection of *B. trehalosi* was more frequent in samples enriched for 10 hours ($p = 0.03$) and 14

hours ($p < 0.001$) than from unenriched samples, but there was no significant difference in detection of *B. trehalosi* between samples enriched for 10 and 14 hours ($p = 0.08$).
